# Medium and short chain fatty acid monoglycerides as an antibiotic alternative reduce diarrhea, improve growth, and modulate gut microbiota in weaned piglets

**DOI:** 10.3389/fvets.2026.1873487

**Published:** 2026-07-21

**Authors:** Yu Li, Yitong Zhou, Chunlian Lu, Hongzhan Cao

**Affiliations:** College of Animal Science and Technology, Agricultural University of Hebei, Baoding, Hebei, China

**Keywords:** antibiotic alternative, diarrhea, growth performance, gut microbiota, weaned piglets

## Abstract

In the global post-antibiotic era, effective alternatives to in-feed antibiotics are urgently needed. This study evaluated a blend of natural molecules of monopropionin, monobutyrin, and monolaurin as an antibiotic alternative in weaned piglets. A total of 180 weaned piglets (Duroc × Landrace × Yorkshire; 24 ± 1 d) were randomly assigned to five dietary treatments for 42 days: control (CON, basal diet), antibiotic (ANTI, basal diet + 500 mg/kg enramycin), and three doses (T1: 600, T2: 1,000, T3: 1,400 mg/kg) of the blend. Growth performance, diarrhea incidence, serum biochemistry, immunity, nutrient digestibility, and fecal microbiota were assessed. Compared with CON, both ANTI and T2 significantly increased final body weight and average daily gain (*p* < 0.01), and reduced feed-to-gain ratio (*p* < 0.05). Diarrhea rate was significantly decreased in ANTI, T2, and T3 (*p* < 0.01). T2 also elevated serum total protein, albumin, and total antioxidant capacity (*p* < 0.05) without increasing alanine aminotransferase activity, and enriched beneficial gut bacteria (*Lactobacillus* and *Eubacterium*) (*p* < 0.05). In contrast, the lowest dose (T1) showed no significant effects on growth or diarrhea, while the highest dose (T3) reduced diarrhea but did not improve growth performance. In conclusion, dietary supplementation with 1,000 mg/kg of this blend effectively improved growth performance, enhanced antioxidant capacity, and modulated gut microbiota in weaned piglets, demonstrating promising potential as an antibiotic alternative.

## 1 Introduction

In modern intensive swine production, the weaning period represents a critical phase during which piglets are subjected to multiple stressors (1). The immaturity of intestinal function, insufficient secretion of digestive enzymes, the withdrawal of maternal antibodies, and abrupt changes in both environment and diet collectively make this period one of the most concentrated windows of stress and immune challenge (2, 3). In practice, this leads to reduced feed intake, growth retardation, and increased diarrhea, which not only compromise subsequent performance but also impair health and can even cause mortality (4, 5). Dietary antibiotics were once widely used to enhance feed efficiency and animal health. However, misuse has led to bacterial resistance and residues, challenging sustainable husbandry and public health (6, 7). Therefore, searching for effective antibiotic alternatives has become a research hotspot (8).

Current alternatives include probiotics, enzymes, herbal medicines, and acidifiers (9–12). They have shown promise but still face issues such as poor stability, high cost, and insufficient specificity (13). Fatty acids and their derivatives, with strong inhibitory effects against common pathogens, represent one of the most promising classes (14). Medium chain (C6–C12) and short chain (C1–C5) fatty acid glycerides are esterification products of glycerol with MCFAs and SCFAs. MCFAs (caproic, caprylic, capric, lauric) possess emulsifying and antimicrobial functions (15). SCFAs (acetic, propionic, butyric) play important roles in immunity, metabolism, and endocrine regulation (16). Due to their shorter carbon chains, they are rapidly digested and absorbed by simple diffusion, bypassing the steps required for long-chain fatty acids (17). Their absorption efficiency is high, even without enzymatic catalysis (18). Once absorbed, they are quickly oxidized within mitochondria, providing rapid energy with minimal tissue deposition (19). As feed additives, these glycerides have shown significant growth-promoting effects. For example, dietary inclusion of 0.20% tributyrin plus 0.15% nucleotides enhanced innate immunity and reduced intestinal damage (20). SCFA glycerides affect immune cell gene expression and function (21). Adding 1.5% medium-chain triglycerides improved intestinal microecology and reduced diarrhea in weaned piglets (22). MCFAs also increase villus height, positively correlated with weight gain (23).

Although individual monoglycerides have been studied (24, 25), systematic evaluation of a ternary blend of propionate, butyrate, and laurate as a complete antibiotic substitute is lacking, and its optimal dose remains undefined. We hypothesized that dietary supplementation with the mixture of compounds would improve growth performance, immune function, and gut health in weaned piglets, with an optimal dose comparable to the antibiotic enramycin. Therefore, this study aimed to determine the optimal inclusion level and evaluate its effects on growth, diarrhea, serum biochemistry, immunity, nutrient digestibility, and fecal microbiota.

## 2 Materials and methods

### 2.1 The mixture of compounds and diets

The mixture of compounds used in this study was commercially obtained from Hebei Shijiazhuang Yunong Breeding Co., Ltd. (Shijiazhuang, China). The product is a blend of three monoglycerides: glycerol monopropionate, glycerol monobutyrate, and glycerol monolaurate, mixed in a 1:1:1 weight ratio. According to the manufacturer’s specifications, the total monoglyceride content is ≥95%, with less than 5% free fatty acids and glycerol. The mixture was incorporated directly into the experimental diets at the designated supplementation levels (600, 1,000, and 1,400 mg/kg). The supplementation levels were selected based on previous studies. Research has demonstrated that supplementation with similar compounds, such as glycerol monolaurate at 1000 mg/kg (24), tributyrin at 500–1,000 mg/kg (25), and a mixture of glycerol monolaurate and tributyrin, significantly improved growth performance and reduced diarrhea incidence in weaned piglets. Among these, the study by Dong et al. used supplementation levels of 1,000 and 2,000 mg/kg, further confirming the efficacy of such doses (26). Therefore, a range of doses covering low (600 mg/kg), medium (1,000 mg/kg), and high (1,400 mg/kg) levels was designed to investigate the dose–response relationship and determine the optimal inclusion level. All diets were formulated to meet the nutrient requirements recommended by NRC (2012), and the ingredient composition and nutrient content of the basal diet are presented in Table 1.

**Table 1:** Ingredients and analyzed chemical composition of the basal diet.

Ingredients	Content (%)	Nutrient	Content (%)
Corn	57.00	DE(MJ/kg)	14.35
Wheat middling	1.00	CP	21.20
Soybean oil	2.40	CF	2.04
Soybean meal	14.00	Ca	0.90
Soy protein isolate	3.70	TP	0.66
Extruded soybean	5.00	AP	0.52
Corn protein meal	4.00	Lys	1.37
Import fishmeal	4.00	Met	0.40
Dried whey	2.00	Thr	0.79
Glucose	2.00		
NaCl	0.30		
Limestone	1.00		
Dicalcium phosphate	1.30		
Premix a Total	2.30 100.00		

^1^DE, digestible energy (calculated value based on NRC, 2012). Premix provided per kg of diet: vitamin A 12,000 IU, vitamin D₃ 3,000 IU, vitamin E 50 IU, vitamin K₃ 3 mg, vitamin B₁ 2 mg, vitamin B₂ 6 mg, vitamin B₆ 4 mg, vitamin B₁₂ 0.03 mg, niacin 30 mg, pantothenic acid 15 mg, folic acid 1 mg, biotin 0.1 mg, Fe 100 mg, Cu 20 mg, Zn 100 mg, Mn 50 mg, I 0.5 mg, Se 0.3 mg.

### 2.2 Animals and experimental design

All experimental procedures were approved by the Laboratory Animals Management and Ethics Committee of Hebei Agricultural University (Approval No. 2026099; Approval Date: January 30, 2026). A total of 180 weaned piglets (Duroc × Landrace × Yorkshire; 24 ± 1 days of age) were randomly assigned (using a randomized complete block design with initial body weight and sex as blocking factors) to 5 groups with 4 replicates per group and 9 piglets per replicate. The trial consisted of five treatment groups: an ANTI group (basal diet + 500 mg/kg enramycin), a CON group (basal diet), and three experimental groups (T1, T2, T3) in which the basal diet was supplemented with the mixture at 600, 1,000, and 1,400 mg/kg, respectively. During the 3-day adaptation period, all piglets were fed the basal diet. On day 0 of the formal trial, piglets were directly switched to their respective experimental diets, which were maintained throughout the 42-day experimental period. The pig housing environment was meticulously controlled: average indoor temperature 24–26 °C, relative humidity 60–70%.

### 2.3 Sample collection

Fresh, uncontaminated fecal samples were collected from each replicate in all groups on the mornings of days 40, 41, and 42 of the trial prior to feeding, sealed in airtight bags. One portion was frozen for apparent digestibility determination, while another portion was thoroughly mixed with hydrochloric acid and then frozen for protein digestibility analysis. All sample collections strictly adhered to the animals’ normal physiological state piglets maintained ad libitum access to feed and water without any fasting intervention to ensure samples genuinely reflected normal digestive and excretory processes under dietary treatments, thereby avoiding metabolic fluctuations induced by fasting that could affect digestibility evaluation. On day 42, fresh fecal samples not in contact with the ground were collected from each replicate. Using sterile toothpicks, the inner core of the samples was extracted, and the fecal material was aliquoted into 2 mL cryovials at 0.5–2 g per tube and stored at −80 °C for fecal microbiota indicator analysis. On day 42 of the trial, after a 12 h fasting period, one piglet from each replicate pen with a body weight closest to the replicate average was selected for blood sampling. A total of 20 piglets (4 replicates × 5 treatment groups) were sampled. Blood samples (approximately 10 mL) were collected via anterior vena cava puncture using sterile syringes. The blood was immediately transferred to pro-coagulant tubes and allowed to stand at room temperature for 2–3 h to clot. Serum was then separated by centrifugation at 1,000 × g for 10 min at 4 °C. The supernatant was aliquoted into 1.5 mL centrifuge tubes and stored at −20 °C until subsequent analysis of serum biochemical parameters and immune indices.

### 2.4 Determination of experimental indicators

#### 2.4.1 Growth performance and diarrhea rate

On days 0 and 42 of the experiment, all piglets were subjected to a 12 h fasting period (with ad libitum access to water) prior to weighing. Feed was withdrawn from the feeders 12 h before the scheduled weighing time to ensure that body weight measurements were not confounded by variations in gut fill. This standardized procedure was applied consistently across all treatment groups to allow for accurate calculation of average daily gain (ADG), average daily feed intake (ADFI), and feed-to-gain ratio (F/G). ADG was calculated as (final body weight – initial body weight)/number of experimental days. ADFI was calculated as (feed offered – feed refusal)/number of experimental days. F/G was calculated as total feed intake/total weight gain. Diarrhea was monitored daily in all groups. The incidence of diarrhea was assessed using a 5-point fecal consistency scoring system, with visual evaluation of fecal characteristics performed each day. The scoring criteria were as follows: 1 point (hard, dry, and pelleted), 2 points (firm and well-formed), 3 points (soft, moist, and retains shape), 4 points (soft and unformed), and 5 points (watery and liquid). A fecal score of 4 or 5 was classified as diarrhea. The diarrhea incidence (%) was calculated as the number of piglets with diarrhea divided by the total number of weaned piglets and expressed as a percentage (27).

#### 2.4.2 Serum biochemical and immune indicators

Serum biochemical indices, including blood urea nitrogen (BUN), triglycerides (TG), total cholesterol (T-CHO), total protein (TP), albumin (ALB), calcium (Ca), phosphorus (P), alanine aminotransferase (ALT), aspartate aminotransferase (AST), alkaline phosphatase (ALP), and malondialdehyde (MDA), were measured using commercial assay kits purchased from the Nanjing Jiancheng Bioengineering Institute. Serum immune parameters, including immunoglobulins (IgA, IgG, IgM), complement components 3 and 4 (C3, C4), and the cytokines interleukin-2 (IL-2) and interleukin-4 (IL-4), were determined using commercial enzyme-linked immunosorbent assay (ELISA) kits purchased from the Nanjing Jiancheng Bioengineering Institute. Serum biochemical indices, including blood urea nitrogen (BUN; Cat. No. C013-2-1), triglycerides (TG; Cat. No. A110-1-1), total cholesterol (T-CHO; Cat. No. A111-1-1), total protein (TP; Cat. No. A045-4-2), albumin (ALB; Cat. No. A028-2-1), calcium (Ca; Cat. No. C004-2-1), phosphorus (P; Cat. No. C006-2-1), alanine aminotransferase (ALT; Cat. No. C009-2-1), aspartate aminotransferase (AST; Cat. No. C010-2-1), alkaline phosphatase (ALP; Cat. No. A059-2-2), and malondialdehyde (MDA; Cat. No. A003-1-2), were measured using commercial assay kits purchased from the Nanjing Jiancheng Bioengineering Institute (Nanjing, China). Serum immune parameters, including immunoglobulins (IgA, Cat. No. H108-1-2; IgG, Cat. No. H106-1-2; IgM, Cat. No. H107-1-2), complement components 3 and 4 (C3, Cat. No. H186-1-2; C4, Cat. No. H187-1-2), and the cytokines interleukin-2 (IL-2, Cat. No. H003-1-2) and interleukin-4 (IL-4, Cat. No. H005-1-2), were determined using commercial enzyme-linked immunosorbent assay (ELISA) kits purchased from the Nanjing Jiancheng Bioengineering Institute (Nanjing, China). All procedures were performed strictly according to the manufacturer’s instructions.

Serum biochemical indices were measured using commercial assay kits (Nanjing Jiancheng Bioengineering Institute, Nanjing, China) with the following catalog numbers: blood urea nitrogen (BUN, C013-2-1), triglycerides (TG, A110-1-1), total cholesterol (T-CHO, A111-1-1), total protein (TP, A045-4-2), albumin (ALB, A028-2-1), calcium (Ca, C004-2-1), phosphorus (P, C006-2-1), alanine aminotransferase (ALT, C009-2-1), aspartate aminotransferase (AST, C010-2-1), alkaline phosphatase (ALP, A059-2-2), and malondialdehyde (MDA, A003-1-2). Serum immune parameters, including immunoglobulins (IgA, H108-1-2; IgG, H106-1-2; IgM, H107-1-2), complement components (C3, H186-1-2; C4, H187-1-2), and cytokines (IL-2, H003-1-2; IL-4, H005-1-2), were determined using commercial ELISA kits from the same manufacturer. All procedures followed the manufacturer’s instructions.

#### 2.4.3 Apparent digestibility

The nutrient composition of the basal diet was analyzed according to standard procedures established by the AOAC (2006). Specifically, crude protein (CP; method 990.03) was determined using a Foss Kjeltec 8,400 fully automated Kjeldahl apparatus, with a nitrogen conversion factor of 6.25 applied in calculations. Ether extract (method 920.39) was analyzed using Soxhlet extraction. Phosphorus and calcium concentrations in the feed were measured by the ammonium molybdate colorimetric method and the EDTA titration method, in compliance with the Chinese national standards GB/T 6437–2018 and GB/T 6436–2018, respectively. Additionally, the metabolizable energy (ME), gross energy (GE), and amino acid content of the diet were calculated according to the Chinese Nutrient Requirements for Swine (NY/T 65-2004).

#### 2.4.4 Fecal microbiota analysis

Total genomic DNA was extracted from fecal samples using the E. Z. N. A.^®^ Stool DNA Kit. DNA concentration and purity were determined using a NanoDrop 2000 spectrophotometer (Thermo Fisher Scientific, Wilmington, DE, United States). The V3–V4 hypervariable region of the bacterial 16S rRNA gene was amplified by PCR using the universal primers 341F (5′-CCTACGGGNGGCWGCAG-3′) and 805R (5′-GACTACHVGGGTATCTAATCC-3′). The PCR reaction mixture (25 μL) contained 12.5 μL of Phusion Hot Start Flex 2X Master Mix, 2.5 μL of each primer (10 μM), 50 ng of DNA template, and nuclease-free water to volume. The PCR conditions were as follows: initial denaturation at 98 °C for 30 s; 35 cycles of denaturation at 98 °C for 10 s, annealing at 54 °C for 30 s, and extension at 72 °C for 45 s; followed by a final extension at 72 °C for 10 min. Each sample was amplified in triplicate. The resulting amplicons were purified using AMPure XT beads (Beckman Coulter Genomics, Danvers, MA, United States) and quantified with a Qubit fluorometer (Invitrogen, USA). After assessing the quality of the amplicon libraries using an Agilent 2100 Bioanalyzer and the Kapa Library Quantification Kit, paired-end sequencing (PE250) was performed on an Illumina NovaSeq platform by LC-Bio Technology Co., Ltd. (Hangzhou, China).

Bioinformatic analysis was conducted as follows: Raw paired-end reads were merged using FLASH. Quality filtering was performed using fqtrim (v0.94) to obtain clean reads. Chimeric sequences were identified and removed using Vsearch (v2.3.4). Amplicon Sequence Variants (ASVs) were inferred, and a feature table along with representative sequences were generated using the DADA2 pipeline. The feature table was normalized prior to diversity analysis. Taxonomic assignment of the representative sequences was performed against the SILVA database (release 132). Alpha diversity indices (Chao1, ACE, Simpson, Shannon, and Coverage) and Beta diversity were calculated using QIIME2, and corresponding plots were generated with R packages. Species classification annotation and differential analysis were performed based on the ASVs.

### 2.5 Statistical analysis

Data were collated using Microsoft Excel (version 2019) and analyzed with SPSS software (version 25.0, IBM Corp., Armonk, NY, United States). Growth performance, serum biochemical parameters, immune indices, nutrient digestibility, and alpha diversity were analyzed by one-way ANOVA followed by Duncan’s multiple range test. Diarrhea incidence was analyzed using a generalized linear model with a logit link. A pooled SEM was calculated for each parameter. Results are presented as mean ± SEM. Statistical significance was set at *p* < 0.05, and extremely significant differences at *p* < 0.01.

## 3 Results

### 3.1 Growth performance and diarrhea

As shown in Table 2, Compared with the CON group, both the ANTI group and T2 showed highly significant increases in final body weight and average daily gain (*p* < 0.01), while T1 and T3 showed no significant differences from the CON group. Regarding average daily feed intake, there were no significant differences among all groups, but the ANTI group and T2 had numerically higher values than the CON group. For the feed-to-gain ratio, the ANTI group and T2 were significantly lower than the CON group, T1, and T3 (*p* < 0.05), whereas T1 and T3 showed no significant differences from the CON group.

**Table 2:** Effects of medium and short chain fatty acid monoglycerides on growth performance of weaned piglets.

Items	CON	ANTI	T1	T2	T3	SEM	*p*-value
IBM (kg)	7.99	8.27	8.22	8.54	8.11	0.27	0.912
FBM (kg)	25.45^B^	28.97^A^	26.62^B^	28.88^A^	25.90^B^	0.66	<0.01
ADG (g)	388.10^B^	451.63^A^	391.37^B^	452.32^A^	383.79^B^	16.56	<0.01
ADFI (g)	955.03^ab^	980.45^a^	931.22^b^	972.86^a^	937.29^b^	3.41	0.067
F/G	2.46^a^	2.17^c^	2.38^a^	2.18^c^	2.44^a^	0.05	0.043

^1^IBM, initial body weight; FBM, final body weight; ADG, average daily gain; ADFI, average daily feed intake; F/G, feed-to-gain ratio. CON, control; ANTI, antibiotic (500 mg/kg enramycin); T1, 600 mg/kg; T2, 1,000 mg/kg; T3, 1,400 mg/kg. Values are means. Pooled SEM and p-values from one-way ANOVA (*n* = 4). In the same row, different lowercase superscript letters indicate significant differences (*P* < 0.05), different uppercase superscript letters indicate significant differences (*P* < 0.01).

Diarrhea rate was significantly reduced in ANTI, T2, and T3 compared with CON (*p* < 0.01), whereas T1 showed no significant difference (Table 3).

**Table 3:** Effects of medium and short chain fatty acid monoglycerides on diarrhea rate in weaned piglets.

Items	CON	ANTI	T1	T2	T3	SEM	*P*-value
Frequency of diarrhea %	2.92^A^	1.71^C^	2.63^A^	1.82^C^	2.04^B^	0.15	<0.01

^1^Values are means. Pooled SEM and P-value derived from generalized linear model (binomial, logit link). In the same row, different lowercase superscript letters indicate significant differences (*P* < 0.05), different uppercase superscript letters indicate significant differences (*P* < 0.01).

### 3.2 Serum biochemical parameters

Table 4 shows that compared with the CON group, TP, ALP, MDA, and T-AOC were significantly increased in all treatment groups (*p* < 0.05); TG and T-CHO were significantly increased in the ANTI and T2 groups (*p* < 0.01); AST and ALT were significantly increased in the ANTI and T3 groups (*p* < 0.01); BUN and P were significantly increased only in the T1 group (*p* < 0.01). No significant differences were found in the remaining indicators compared with the CON group.

**Table 4:** Effects of medium and short chain fatty acid monoglycerides on serum biochemical parameters in weaned piglets.

Items	CON	ANTI	T1	T2	T3	SEM	*P*-value
TP (g/L)	46.14^C^	63.71^Aa^	62.09^Aa^	58.33^ab^	56.38^b^	2.00	<0.01
ALB (g/L)	36.17^C^	39.59^Aa^	33.8^D^	37.69^ab^	34.74^CD^	0.74	<0.01
TG (nmol/L)	0.32^C^	0.57^A^	0.38^b^	0.5^A^	0.31^C^	0.02	<0.01
T-CHO (nmol/L)	0.24^C^	0.33^Ab^	0.29^bC^	0.37^A^	0.26^bC^	0.03	<0.01
BUN (nmol/L)	1.81^b^	2.03^b^	2.87^A^	2.25^b^	2.14^b^	0.19	<0.01
AST (U/L)	145.75^bC^	158.35^Aa^	141.15^C^	147.66^bC^	153.06^ab^	3.94	<0.01
ALT (U/L)	140.64^Bb^	153.8^Aa^	143.57^b^	137.72^Bb^	152.67^Aa^	3.12	<0.01
ALP (U/L)	151.05^C^	198.37^Bb^	245.06^Aa^	233.72^Aa^	169.69^C^	7.37	<0.01
MDA (nmol/L)	3.21^D^	5.21^Aa^	4.04^C^	4.37^bC^	4.66^Bb^	0.19	<0.01
P (nmol/L)	2.78^b^	3.52^a^	3.39^a^	3.12^ab^	3.18^ab^	0.17	0.08
Ca(nmol/L)	1.59^ab^	1.73^Aa^	1.45^Bb^	1.47^Bb^	1.61^ab^	0.07	0.02
T-AOC (Mm)	0.15^b^	0.24^a^	0.25^a^	0.28^a^	0.24^a^	0.03	0.01

^1^TP, total protein; ALB, albumin; TG, triglyceride; T-CHO, total cholesterol; BUN, blood urea nitrogen; AST, aspartate aminotransferase; ALT, alanine aminotransferase; ALP, alkaline phosphatase; MDA, malondialdehyde; P, phosphorus; Ca, calcium; T-AOC, total antioxidant capacity. CON, control; ANTI, antibiotic; T1–T3 as in Table 2. Values are means. Pooled SEM and *P*-values from one-way ANOVA (*n* = 4). In the same row, different lowercase superscript letters indicate significant differences (*P* < 0.05), different uppercase superscript letters indicate significant differences (*P* < 0.01).

### 3.3 Serum immune parameters

As shown in Table 5, Compared with the CON group, the levels of IgM and IgG in T3 were significantly decreased (*p* < 0.01), while no significant differences were observed among the other groups, although T1 and T2 showed an increasing trend. The IL-2 level in T1 was significantly decreased compared with the CON group (*p* < 0.01), with no significant differences among the other groups. The C3 level in T3 was significantly decreased compared with the ANTI group (*p* < 0.01). No significant differences were observed in IgA and C4 among all groups (*p* > 0.05).

**Table 5:** Effects of medium and short chain fatty acid monoglycerides on serum immune parameters in weaned piglets.

Items	CON	ANTI	T1	T2	T3	SEM	*P*-value
IgM (μg/mL)	8.5^ab^	8.26^ab^	8.82^a^	9.13^A^	7.64^B^	0.37	<0.01
IgG (μg/mL)	149.15^A^	148.89^A^	126.78^ab^	134.41^ab^	117.51^B^	7.43	<0.01
IgA (μg/mL)	29.05	29.74	27.96	27.85	27.56	1.48	0.80
IL-2 (pg/mL)	756^A^	759.39^A^	611.77^B^	769.01^A^	826.41^A^	26.13	<0.01
IL-4 (pg/mL)	304^ab^	278.49^B^	310.12^ab^	342.19^A^	317.6^AB^	16.33	0.10
C3(μg/mL)	6.93^B^	8.22^A^	6.55^B^	7.4^ab^	6.74^B^	0.30	<0.01
C4(μg/mL)	73.41	76.00	67.95	69.86	75.76	0.61	0.34

^1^IgM, immunoglobulin M; IgG, immunoglobulin G; IgA, immunoglobulin A; IL-2, interleukin-2; IL-4, interleukin-4; C3, complement component 3; C4, complement component 4. CON, control; ANTI, antibiotic; T1–T3 as in Table 2. Values are means. Pooled SEM and P-values from one-way ANOVA (*n* = 4). In the same row, different lowercase superscript letters indicate significant differences (*P* < 0.05), different uppercase superscript letters indicate significant differences (*P* < 0.01).

### 3.4 Nutrient digestibility

Table 6 shows that compared with the CON group, no significant differences were observed in the digestibility of GE, Ca, or P among any of the treatment groups (*p* > 0.05). Regarding EE digestibility, the ANTI group was significantly lower than T1, T2, and T3 (*p* < 0.05). For CP digestibility, the T3 group was significantly higher than the T1, T2, and ANTI groups (*p* < 0.05). No other significant differences were found among the remaining groups.

**Table 6:** Effects of medium and short chain fatty acid monoglycerides on nutrient digestibility in weaned piglets.

Items	CON	ANTI	T1	T2	T3	SEM	*P*-value
GE	81.24	78.67	83.08	76.76	77.84	0.42	0.06
EE	77.59^ab^	75.29^b^	79.55^a^	81.7^a^	78.71^a^	0.48	0.02
CP	78.79^ab^	74.43^b^	76.69^b^	73.63^b^	82.89^a^	0.52	0.01
Ca	73.22	75.16^a^	72.73	75.78	74.66	0.39	0.31
*P*	70.66	69.83	72.17	72.67	70.72	0.34	0.20

^1^GE, gross energy; EE, ether extract; CP, crude protein; Ca, calcium; P, phosphorus. CON, control; ANTI, antibiotic; T1–T3 as in Table 2. Values are means. Pooled SEM and *p*-values from one-way ANOVA (*n* = 4). In the same row, different lowercase superscript letters indicate significant differences (*P* < 0.05), different uppercase superscript letters indicate significant differences (*P* < 0.01).

### 3.5 Fecal microbiota

**Figure 1:**
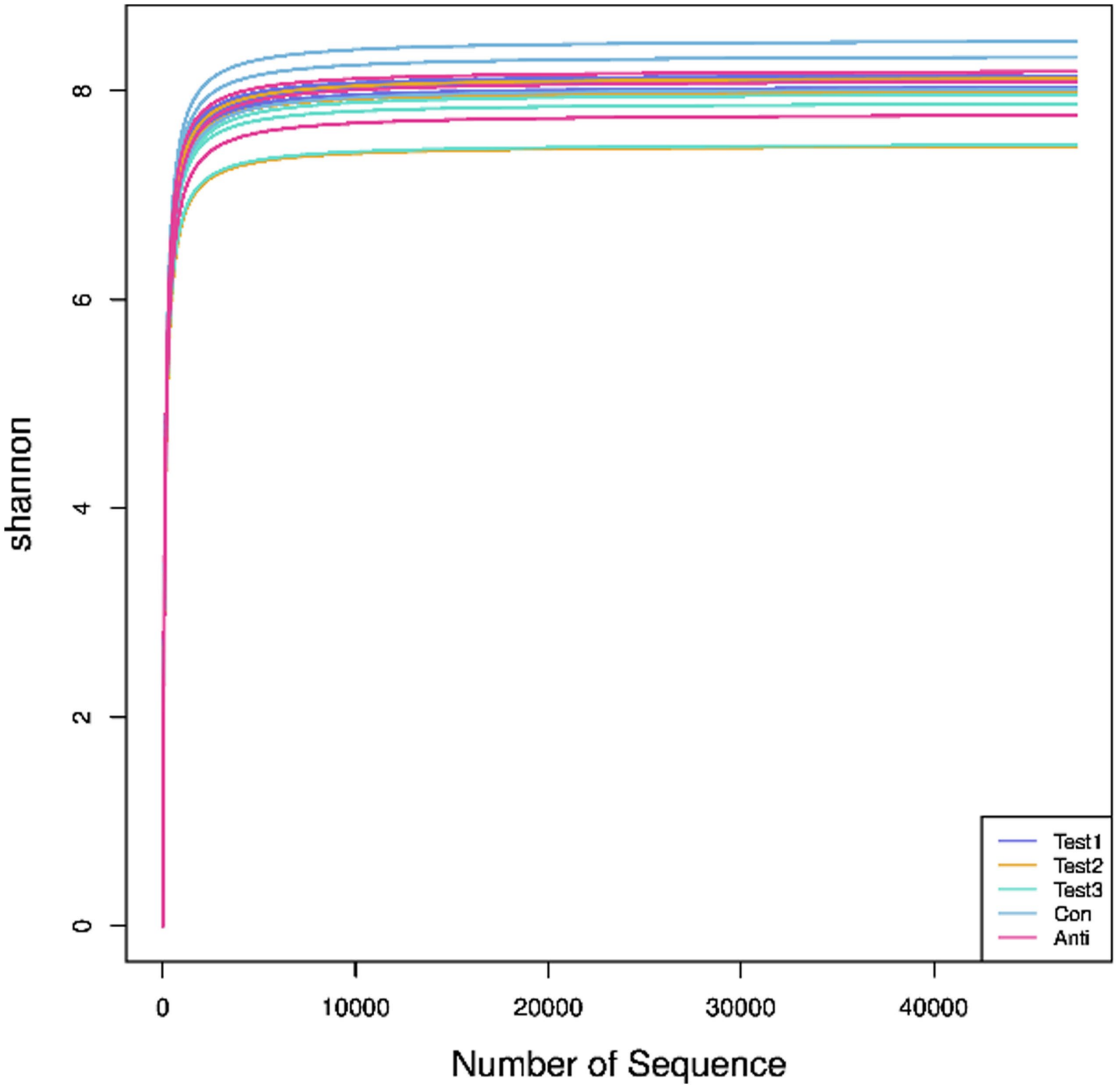
Sample dilution curve.

**Figure 2:**
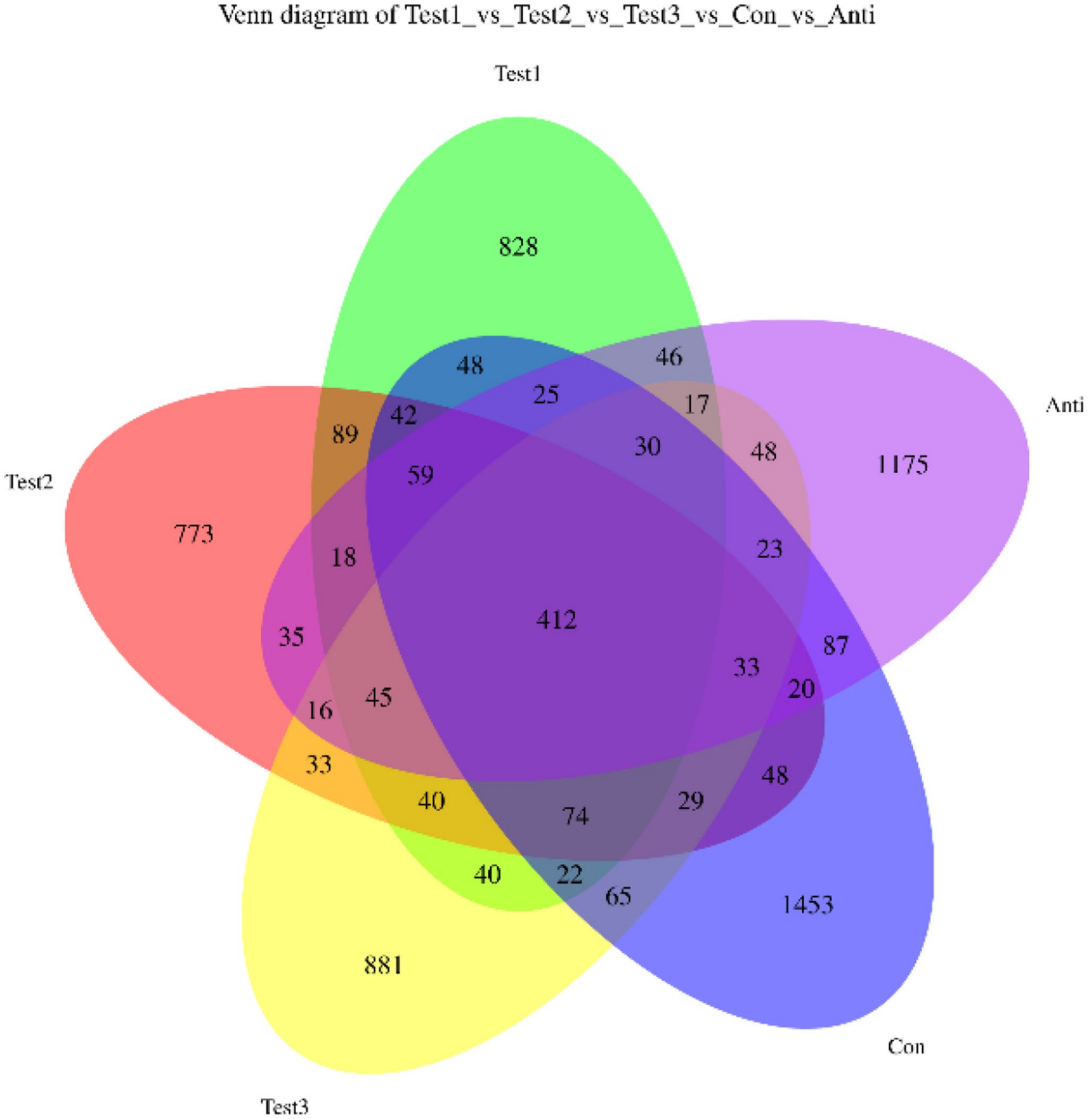
Representation of the OTUs between different groups via Venn diagram.

High-throughput sequencing yielded 1,054,210 high-quality sequences and 13,928 OTUs. Rarefaction curves approached saturation (Figure 1). Venn analysis showed 412 core OTUs shared by all groups (2.96% of total). The CON group had the highest number of unique OTUs (1,453, 10.43%), followed by ANTI (1,175, 8.44%) (Figure 2).

As shown in Table 7, Compared with the CON group, no significant differences were observed in Chao1 index among CON, ANTI, and T1, while ANTI was significantly higher than T2 and T3 (*p* < 0.05). For the Shannon index, ANTI was significantly higher than T3 (*p* < 0.05), and no other significant differences were found among the remaining groups.

**Table 7:** Effects of medium and short chain fatty acid monoglycerides on alpha diversity of fecal microbiota in weaned piglets.

Items	CON	ANTI	T1	T2	T3	SEM	*P*-value
Chao1	951.13^ab^	1097.89^a^	918.12^ab^	861.06^b^	829.31^b^	31.19	0.01
Shannon	8^ab^	8.26^a^	8.09^ab^	7.86^ab^	7.76^b^	0.09	0.01

^1^Chao1, richness estimator; Shannon, diversity index. CON, control; ANTI, antibiotic; T1–T3 as in Table 2. Values are means. Pooled SEM and *p*-values from one-way ANOVA (*n* = 4). In the same row, different lowercase superscript letters indicate significant differences (*P* < 0.05), different uppercase superscript letters indicate significant differences (*P* < 0.01).

**Figure 3:**
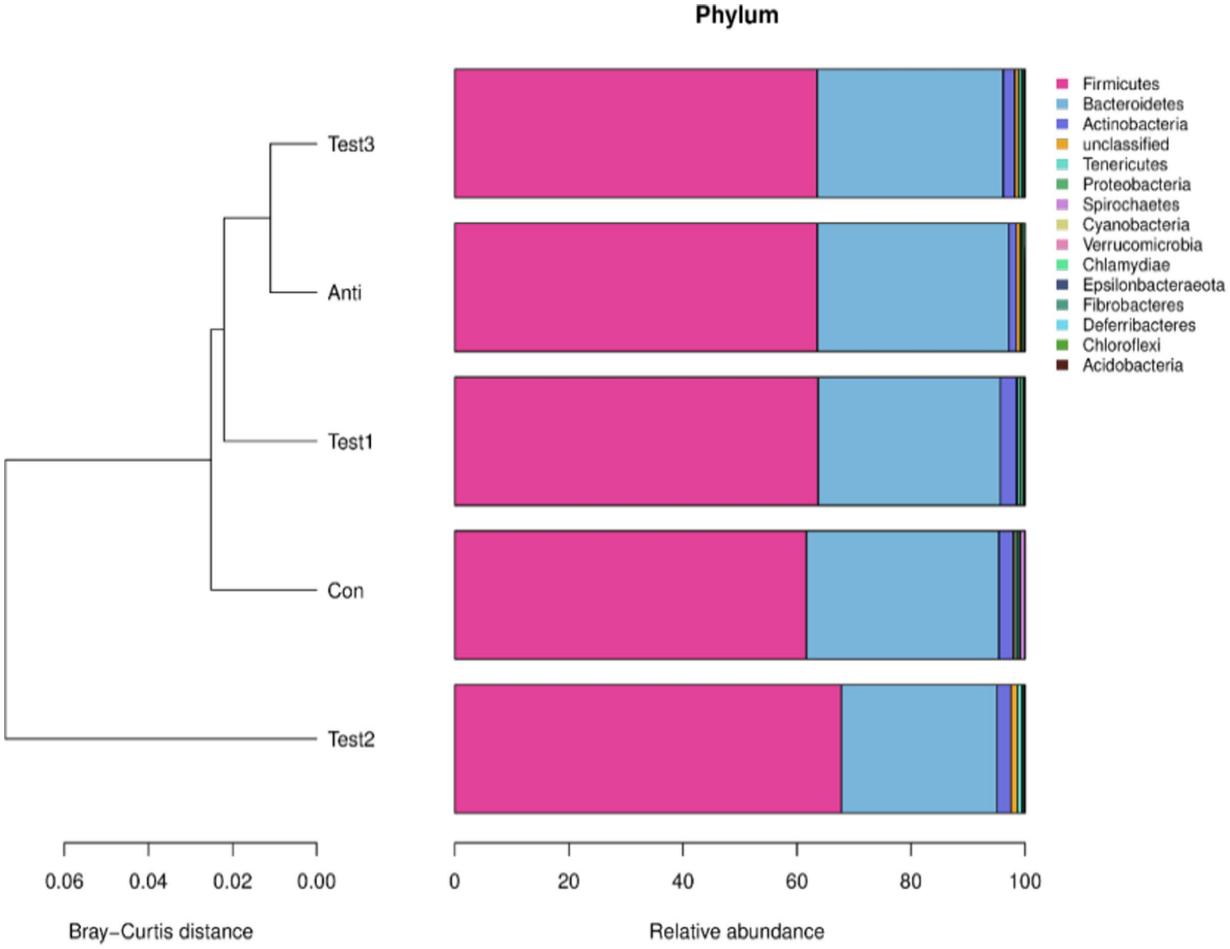
Effects of short chain fatty acid monoglycerides on the level of fecal microbiota in weaned piglets.

**Figure 4:**
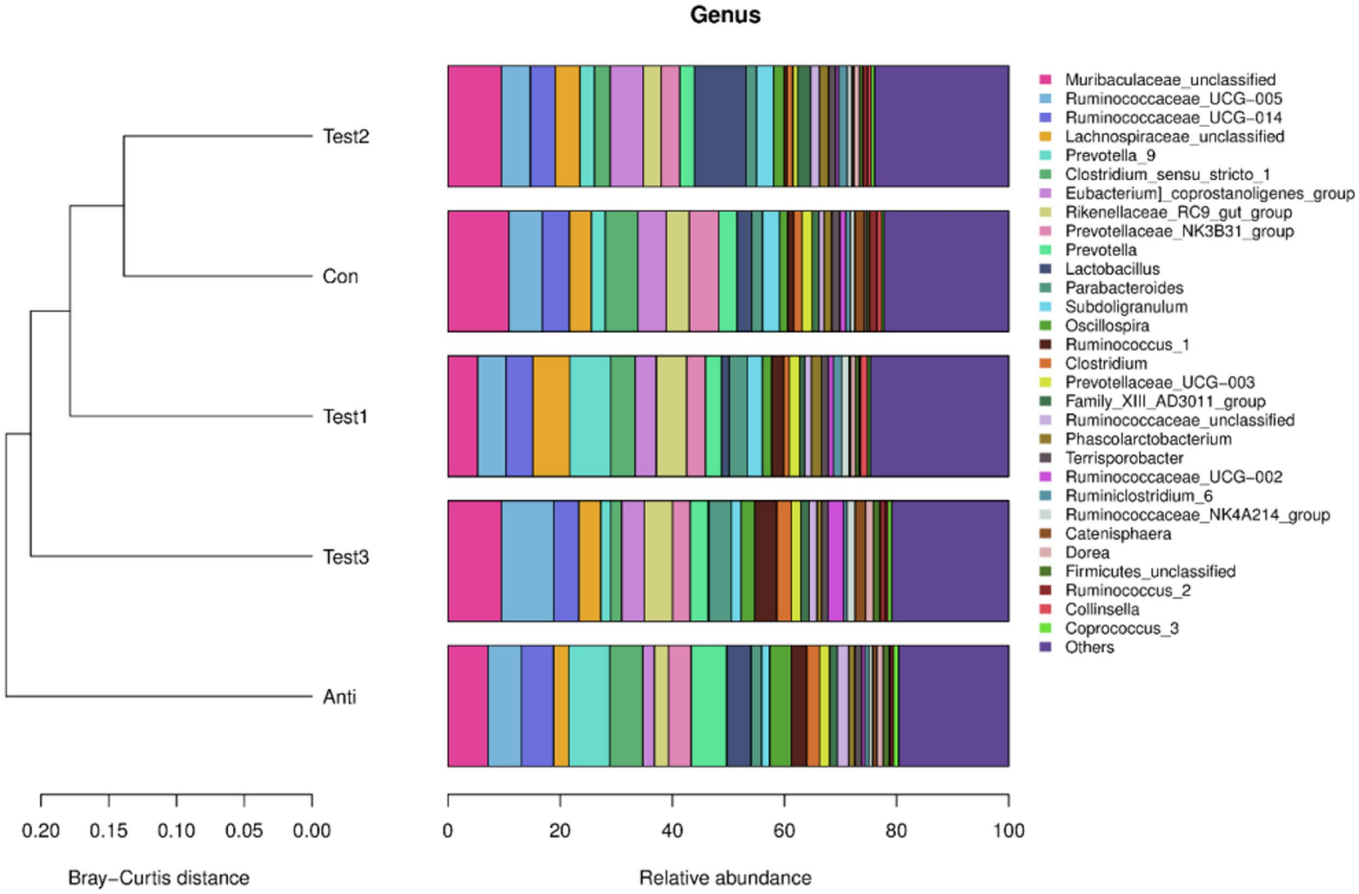
Effects of short chain fatty acid monoglycerides on the level of fecal microbes in weaned piglet.

At the phylum level, Firmicutes and Bacteroidota dominated all groups, with T2 showing the highest Firmicutes abundance (Figure 3). At the genus level, T2 had significantly higher relative abundances of Lactobacillus and Eubacterium than other groups (*p* < 0.05), while abundances of Prevotella and Clostridium were lower (Figure 4).

**Figure 5:**
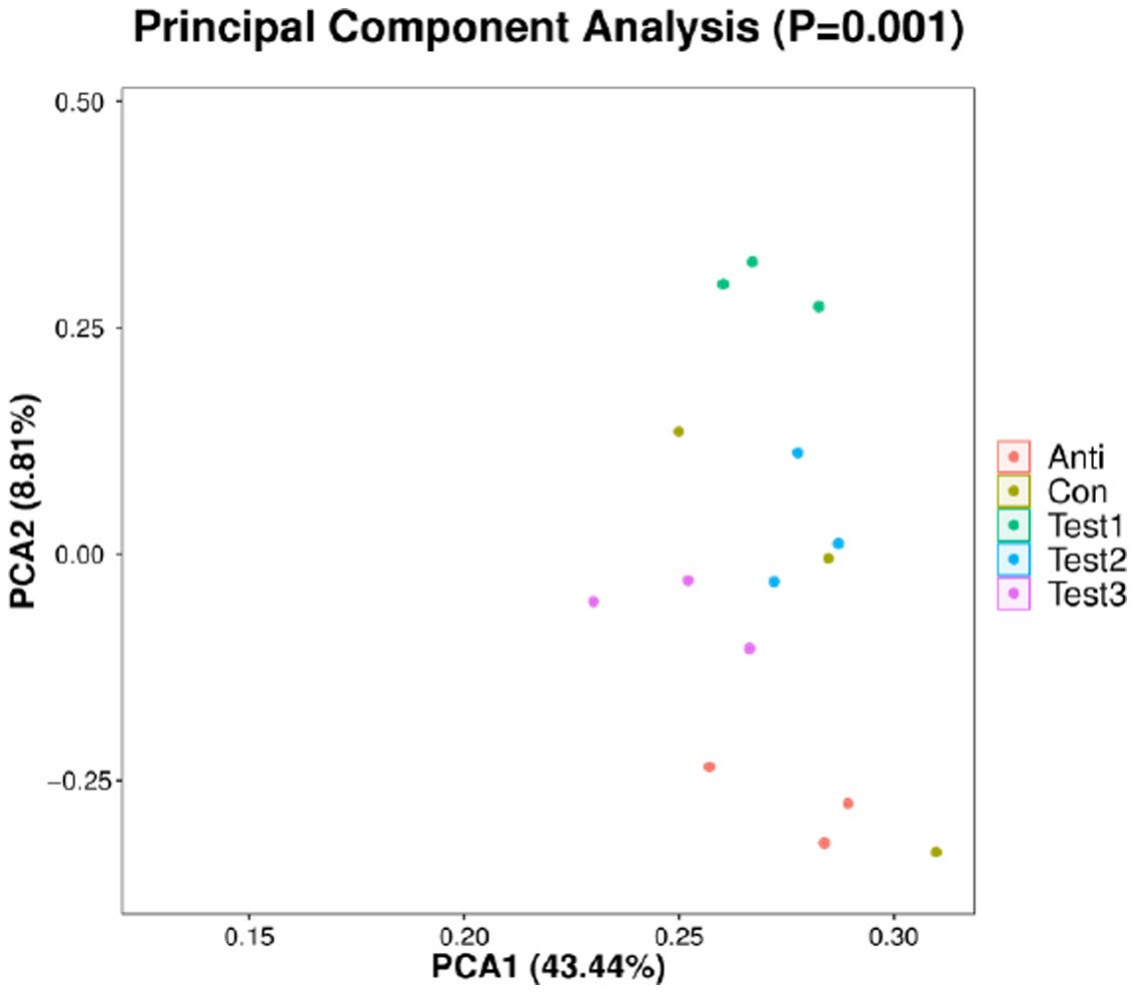
PCA diagram of fecal microbial OUT levels in different treatment groups.

**Figure 6:**
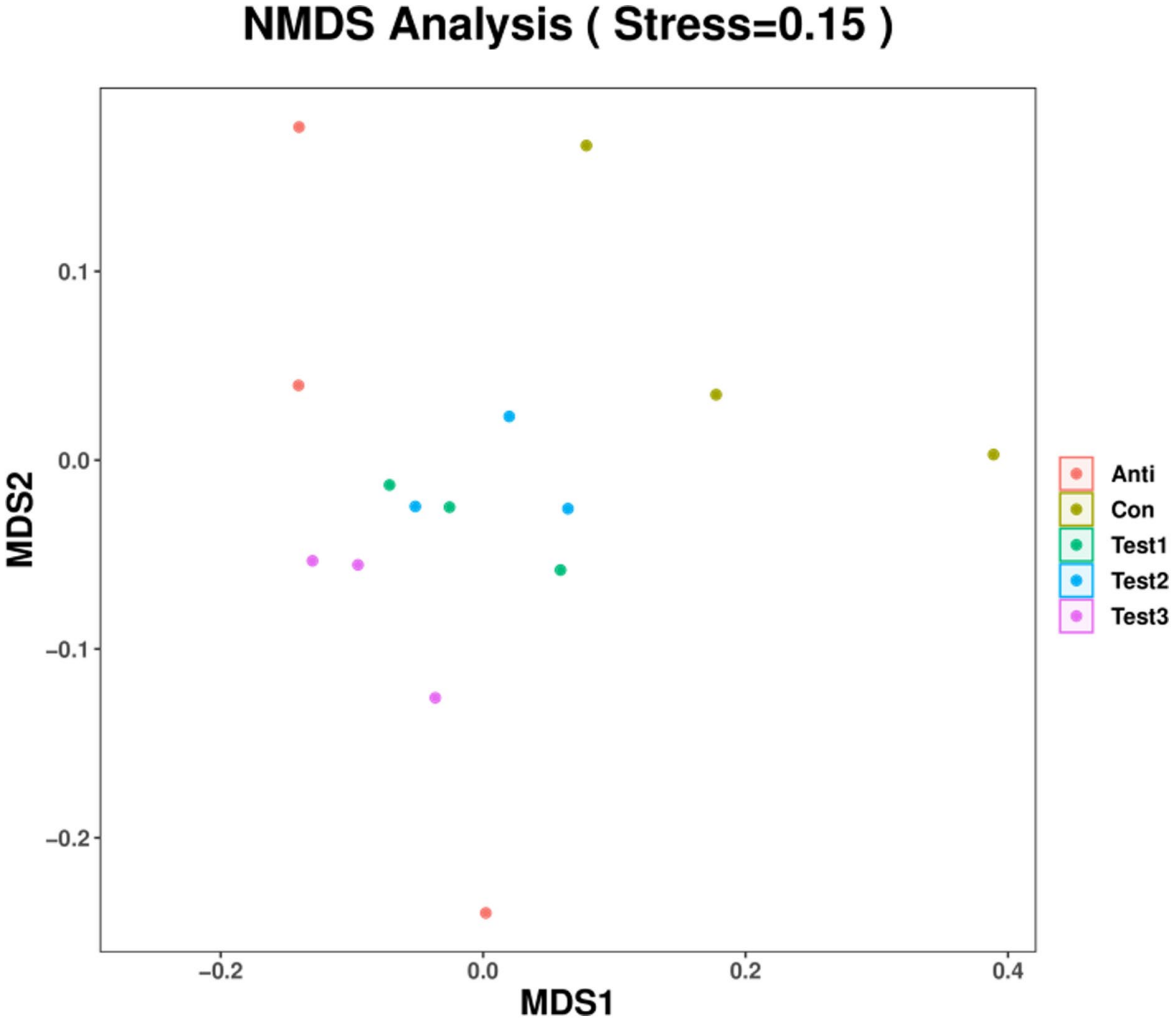
NMDS analysis of fecal microorganisms in different treatment groups.

Principal component analysis (PCA) (Figure 5) showed clear separation between ANTI and the experimental groups. Non-metric multidimensional scaling (NMDS) (Figure 6) confirmed that all experimental groups clustered closely together, indicating similar microbial modulation patterns.

## 4 Discussion

Against the backdrop of the global post-antibiotic era, the development of safe and effective antibiotic alternatives has become critically important. Medium- and short-chain fatty acid monoglycerides are hydrolyzed into organic acids in the animal intestine, where they serve as a rapid energy source and enhance the digestive utilization of fat (28). The results of this study demonstrate that dietary supplementation with a composite monoglyceride blend significantly improved piglet growth performance, nutrient digestibility, serum biochemical parameters, and intestinal microbiota structure, with the T2 group showing the best overall effects. The T2 group exhibited significantly increased average daily gain and final body weight, as well as a significantly reduced feed-to-gain ratio, with growth-promoting effects comparable to those of the antibiotic group. This indicates that the composite monoglyceride blend has good potential as an antibiotic substitute at an appropriate dosage.

Notably, this study revealed a classic inverted U-shaped dose response: the 600 mg/kg dose (T1) failed to exert full efficacy, while the 1,400 mg/kg dose (T3) did not lead to further improvement but rather showed a decline in certain parameters. This suggests that the composite monoglyceride blend has a clear optimal dosage window, and higher doses do not necessarily yield better outcomes. At the low dose, the ability to persistently lower intestinal pH, inhibit pathogenic bacterial proliferation, and provide sufficient energy to intestinal epithelial cells was insufficient, thus failing to achieve the desired effects (29). For the high-dose group, the decline in growth performance was not primarily attributable to poor feed palatability: the T2 group showed a trend toward increased average daily feed intake compared to the control group, indicating that the additive at the appropriate dose does not impair palatability but may instead have a certain palatability enhancing effect. Although ADFI in the T3 group was slightly lower than that in T2, the difference was not statistically significant, suggesting that changes in feed intake alone cannot explain the growth performance decline. More importantly, serum ALT and AST activities were significantly elevated in the T3 group, indicating that high-dose supplementation may increase the hepatic metabolic burden and induce mild hepatocyte stress. Under high-dose conditions, medium-chain fatty acids (MCFAs, e.g., lauric acid) released from monoglyceride hydrolysis in the gut are primarily transported via the portal vein to the liver for *β*-oxidation (30), whereas short-chain fatty acids (SCFAs, e.g., butyric acid) are preferentially utilized by intestinal epithelial cells (31). Thus, the increased hepatic metabolic load under high-dose conditions mainly originates from excessive β-oxidation of MCFAs rather than a direct effect of SCFAs. Lauric acid (C12:0) undergoes rapid β-oxidation in hepatic mitochondria, primarily via medium- and long-chain acyl-CoA dehydrogenases, generating substantial acetyl-CoA that enters the tricarboxylic acid (TCA) cycle. When the intake exceeds the oxidative capacity of the liver, this may lead to the accumulation of metabolic intermediates and oxidative stress, subsequently increasing hepatocyte membrane permeability (manifested as elevated ALT and AST) and potentially imposing a metabolic burden on the liver at high doses (32). Accordingly, although the T3 group showed a certain improvement in crude protein digestibility, the accompanying hepatic metabolic pressure and stress response may have counteracted some nutritional benefits, ultimately preventing further improvement in growth performance. Consistent with this, T3 also exhibited decreased levels of immunoglobulin G, immunoglobulin M, and complement C3, suggesting that immune homeostasis was compromised under high-dose conditions.

The T2 group achieved the best total antioxidant capacity and also showed favorable immune indicators. In contrast, the T3 group displayed decreased levels of IgG, IgM, and complement C3, indicating a trend toward immunosuppression, further supporting the notion that the body was in a state of metabolic stress at the high dose. Moreover, diarrhea incidence decreased in all supplementation groups, which can be attributed to the organic acids produced by the composite monoglyceride blend that possess antibacterial and gut-protective effects (33, 34); the T2 group had the lowest diarrhea incidence, further confirming that 1,000 mg/kg is the optimal dosage balancing growth performance, liver safety, and immune function.

Serum total protein did not show a linear dose–response relationship. The T1 group had the highest TP but the poorest growth performance and concurrently elevated blood urea nitrogen, suggesting that nitrogen metabolism efficiency did not improve synchronously at this dose; higher plasma protein may not reflect greater nutrient utilization efficiency, as blood urea nitrogen is a marker of protein requirement and amino acid utilization status (35). By contrast, the T2 group had a moderate TP level yet achieved the best growth performance, with BUN maintained within the normal range, indicating that this dose is more conducive to the deposition and conversion of dietary protein into tissue protein. The T3 group had the lowest TP, accompanied by elevated ALT and AST, further suggesting that high-dose conditions may impair hepatic synthetic function, and that liver stress may compromise protein synthesis (36).

Regarding oxidative stress markers, the T3 group had the highest malondialdehyde level, whereas the T2 group exhibited the best T-AOC, indicating that an appropriate dose helps enhance antioxidant defense, whereas an excessive dose may increase lipid peroxidation (37). Elevated MDA does not necessarily correlate with a higher growth rate; rather, it more directly reflects the degree of lipid oxidative damage and the level of metabolic stress. Therefore, the increased MDA in T3 is more likely associated with the metabolic burden and oxidative stress induced by the high dose, given that the actual growth performance of this group was not superior to that of T2. Albumin and calcium were significantly increased only in the antibiotic group, whereas Ca decreased in T1 and T2; this effect may relate to altered mineral absorption (38). In contrast, BUN and phosphorus increased in T1, which may be a transient effect at the lowest dose and not involve mineral absorption (39).

The improved crude fat digestibility in all supplemented groups (especially T2) confirms that this composite monoglyceride blend acts as an emulsifier and lipid absorption enhancer. This effect is primarily attributable to the emulsifying capacity of lauric acid monoglyceride. As a bio-surfactant, lauric acid monoglyceride reduces oil–water interfacial tension, promotes fat emulsification and dispersion, facilitates lipolysis and subsequent lipid absorption and chylomicron assembly, ultimately improving fat digestion efficiency (40). By comparison, the primary roles of propionic and butyric acid monoglycerides are more likely to involve local acidification, antibacterial activity, and energy provision to intestinal epithelium, with relatively limited emulsifying effects (41). Thus, the improvement in crude fat digestibility observed in this study, particularly the growth performance enhancement in T2, increased dietary energy utilization by improving fat digestion and absorption efficiency. Crude protein digestibility improved only in T3, but this change did not translate into higher plasma TP or better growth performance. This indicates that improved protein digestibility does not necessarily equate to improved protein deposition efficiency, as the latter is also influenced by hepatic synthetic capacity, nitrogen metabolic status, and systemic stress levels. Considering the elevated ALT and AST and declined immune indicators in T3, it can be inferred that although the high-dose treatment improved certain nutrient digestibility parameters, it may have concurrently imposed a higher metabolic burden, thereby attenuating its positive contribution to growth performance.

Microbiota analysis revealed that, compared with the control group, the T2 group showed significant enrichment of *Lactobacillus* and *Eubacterium*, along with a decreased abundance of potentially harmful bacteria, suggesting that the appropriate dose is more conducive to establishing a healthy intestinal microecological structure. *Lactobacillus* can maintain an acidic luminal environment and inhibit pathogen proliferation through acid production (42) while *Eubacterium* includes several well-characterized butyrate-producing species; butyrate not only serves as an important energy source for intestinal epithelial cells but also helps maintain intestinal mucosal barrier integrity (43). The increase in these beneficial genera and the reduction in potential pathogens may be attributed to the organic acids released from monoglyceride hydrolysis, which lower intestinal pH and selectively inhibit Gram-negative bacteria (44). Propionic and butyric acids are weak organic acids, and extensive research has demonstrated their ability to lower intestinal pH, improve nutrient digestibility, and shift the intestinal microbiota toward acid-tolerant bacteria (29, 45). Dietary supplementation with butyrate or a mixture of butyrate and MCFAs has been shown to significantly reduce the pH of intestinal contents (46). Zhang et al. reported that combining sodium butyrate and oregano oil in the diet of weaned piglets increased *Lactobacillus* abundance, which is consistent with the microbial changes observed in the present study (47) Mroz et al. showed that adding organic acids to a diet with high buffering capacity increased the apparent ileal digestibility of crude protein by 2.9–5.9 percentage points. Feeding a low-buffering diet without organic acids increased urine acidity by 1.6 pH units and reduced manure ammonia emissions by 44% (48). In this study, the improvements in crude protein and crude fat digestibility, as well as the favorable changes in intestinal microbiota, are fully consistent with these well-established mechanisms. Therefore, extensive indirect evidence supports that this composite monoglyceride blend acts, at least in part, through intestinal acidification. Future studies should include direct measurements of fecal pH and ammonia content for further validation. Diversity analysis showed that *α*-diversity decreased in the T3 group, suggesting that the high dose may perturb the microbial community structure; whereas the T2 group maintained relatively stable microbial diversity, indicating that the appropriate dose enriches beneficial bacteria without disrupting the overall microbial balance. Principal component analysis and non-metric multidimensional scaling results showed that the microbial community structures of the supplemented groups were clearly separated from that of the antibiotic group, and T2 and T3 exhibited overlapping community structures, indicating that the composite monoglyceride blend can consistently modulate the intestinal microbiota composition, but the intensity of its biological effects varies with dosage (49).

## 5 Conclusion

In summary, the composite monoglyceride blend promoted the growth of weaned piglets by improving crude fat digestibility, modulating the intestinal microbiota, reducing diarrhea incidence, and maintaining favorable antioxidant and immune homeostasis. The emulsifying effect of lauric acid monoglyceride may be the primary mechanism for promoting fat digestion, while the local acidification effect mediated by propionic and butyric acid monoglycerides at the appropriate dose contributes to optimizing the intestinal environment. Specifically, dietary supplementation with 1,000 mg/kg of the compound medium- and short-chain fatty acid monoglyceride preparation (glycerol monopropionate, monobutyrate, and monolaurate) significantly improved growth performance (increased final body weight and average daily gain, reduced feed-to-gain ratio) and decreased diarrhea incidence in weaned piglets, with effects comparable to the antibiotic enramycin. It also enhanced serum antioxidant capacity (T-AOC) and protein metabolism (TP, ALB) without increasing liver enzyme activities, and enriched beneficial gut bacteria (*Lactobacillus* and *Eubacterium*). It should be noted that this composite monoglyceride blend has a clear dosage window, and excessive supplementation may increase the hepatic metabolic burden and diminish the overall benefits. Therefore, under the conditions of this study, 1,000 mg/kg is the recommended optimal supplementation level. However, this study has limitations: the sample size for microbiota analysis was modest (*n* = 4 per group), the trial was conducted under optimal experimental conditions rather than commercial farm settings, and the duration was limited to the nursery phase. Future studies with larger sample sizes, field validation, and longer observation periods are needed to confirm the potential of this preparation as a practical antibiotic alternative.

## Data Availability

The original contributions presented in the study are included in the article/Supplementary material, further inquiries can be directed to the corresponding author/s.

## Glossary

MCFA: medium chain fatty acids
SCFA: short chain fatty acids
ADFI: average daily feed intake
ADG: average daily gain
GE: gross energy
CP: crude protein
EE: ether extract
TP: total protein
ALB: albumin
TG: triglycerides
T-CHO: total cholesterol
BUN: blood urea nitrogen
AST: aspartate aminotransferase
ALT: alanine aminotransferase
ALP: alkaline phosphatase
MDA: malondialdehyde
T-AOC: total antioxidant capacity
IgA: immunoglobulin A
IgG: immunoglobulin G
IgM: immunoglobulin M
IL-2: interleukin-2
IL-4: interleukin-4
C3: complement component 3
C4: complement component 4
